# Expanding the Physiological Role of Aryl-Alcohol Flavooxidases as Quinone Reductases

**DOI:** 10.1128/aem.01844-22

**Published:** 2023-05-08

**Authors:** Patricia Ferreira, Juan Carro, Beatriz Balcells, Angel T. Martínez, Ana Serrano

**Affiliations:** a Departamento de Bioquímica y Biología Molecular y Celular, Facultad de Ciencias, Universidad de Zaragoza, Zaragoza, Spain; b Instituto de Biocomputación y Física de Sistemas Complejos, BIFI (GBsC-CSIC Joint Unit), Universidad de Zaragoza, Zaragoza, Spain; c Centro de Investigaciones Biológicas “Margarita Salas”, CSIC, Madrid, Spain; University of Nebraska-Lincoln

**Keywords:** aryl-alcohol oxidase (AAO), flavooxidases, GMC superfamily, *Pleurotus* AAO, *Bjerkandera adusta* AAO, quinone reduction, heterologous expression, lignocellulose decay

## Abstract

Aryl-alcohol oxidases (AAOs) are members of the glucose-methanol-choline oxidase/dehydrogenase (GMC) superfamily. These extracellular flavoproteins have been described as auxiliary enzymes in the degradation of lignin by several white-rot basidiomycetes. In this context, they oxidize fungal secondary metabolites and lignin-derived compounds using O_2_ as an electron acceptor, and supply H_2_O_2_ to ligninolytic peroxidases. Their substrate specificity, including mechanistic aspects of the oxidation reaction, has been characterized in Pleurotus eryngii AAO, taken as a model enzyme of this GMC superfamily. AAOs show broad reducing-substrate specificity in agreement with their role in lignin degradation, being able to oxidize both nonphenolic and phenolic aryl alcohols (and hydrated aldehydes). In the present work, the AAOs from Pleurotus ostreatus and Bjerkandera adusta were heterologously expressed in Escherichia coli, and their physicochemical properties and oxidizing abilities were compared with those of the well-known recombinant AAO from *P. eryngii.* In addition, electron acceptors different from O_2_, such as *p*-benzoquinone and the artificial redox dye 2,6-Dichlorophenolindophenol, were also studied. Differences in reducing-substrate specificity were found between the AAO enzymes from *B. adusta* and the two *Pleurotus* species. Moreover, the three AAOs oxidized aryl alcohols concomitantly with the reduction of *p*-benzoquinone, with similar or even higher efficiencies than when using their preferred oxidizing-substrate, O_2_.

**IMPORTANCE** In this work, quinone reductase activity is analyzed in three AAO flavooxidases, whose preferred oxidizing-substrate is O_2_. The results presented, including reactions in the presence of both oxidizing substrates—benzoquinone and molecular oxygen—suggest that such aryl-alcohol dehydrogenase activity, although less important than its oxidase activity in terms of maximal turnover, may have a physiological role during fungal decay of lignocellulose by the reduction of quinones (and phenoxy radicals) from lignin degradation, preventing repolymerization. Moreover, the resulting hydroquinones would participate in redox-cycling reactions for the production of hydroxyl free radical involved in the oxidative attack of the plant cell-wall. Hydroquinones can also act as mediators for laccases and peroxidases in lignin degradation in the form of semiquinone radicals, as well as activators of lytic polysaccharide monooxygenases in the attack of crystalline cellulose. Moreover, reduction of these, and other phenoxy radicals produced by laccases and peroxidases, promotes lignin degradation by limiting repolymerization reactions. These findings expand the role of AAO in lignin biodegradation.

## INTRODUCTION

**FIG 1:**
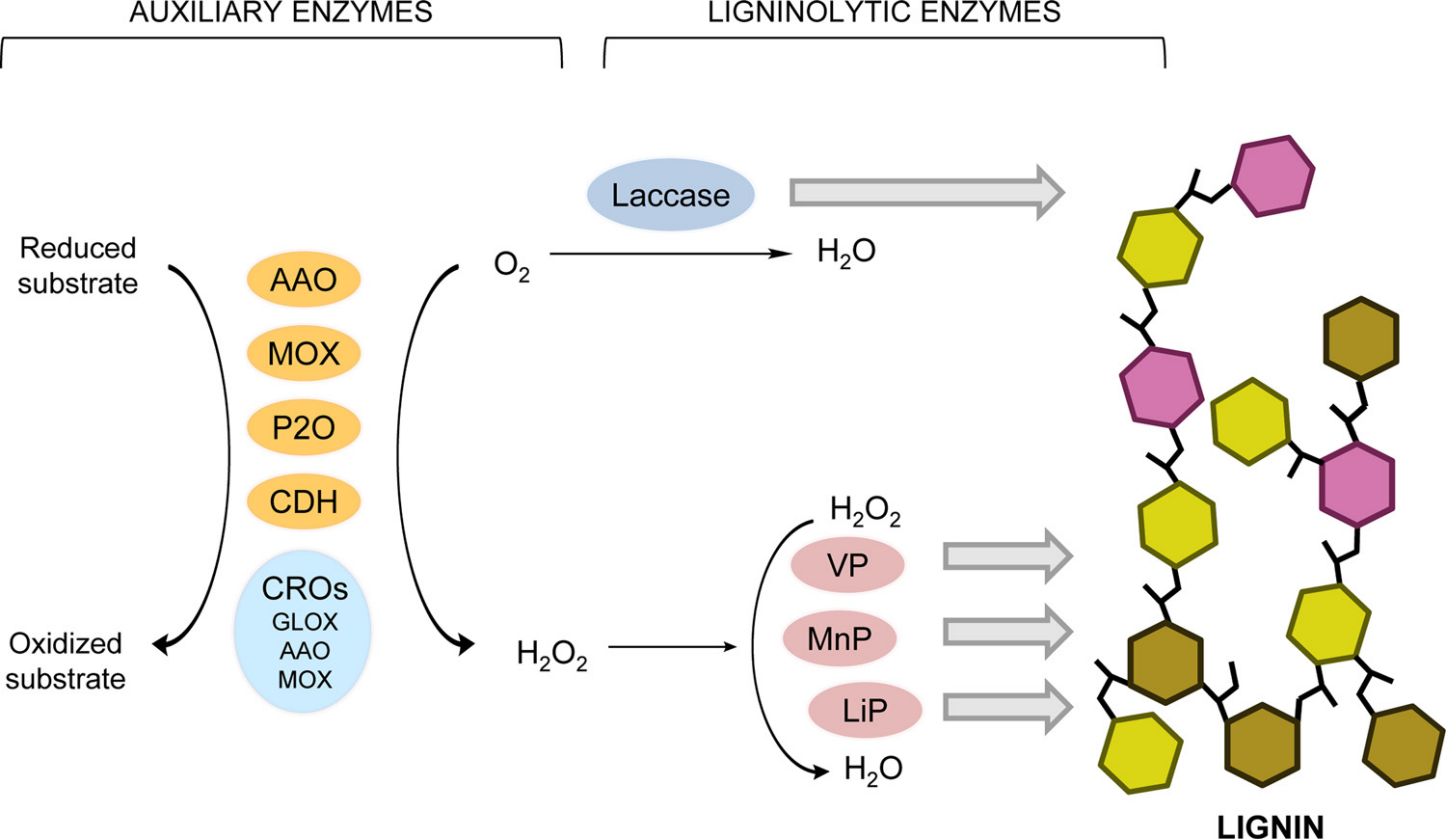
Scheme for the involvement of GMC (and CRO) oxidoreductases as auxiliary enzymes in lignin biodegradation. Oxidation of substrates by auxiliary enzymes releases H_2_O_2_ that is used by heme peroxidases to oxidize lignin (together with laccases using O_2_). GMCs include aryl-alcohol oxidase (AAO), methanol oxidase (MOX), pyranose 2-oxidase (P2O), and cellobiose dehydrogenase (CDH), among other enzyme families. CROs include glyoxal oxidase (GLOX) and recently described counterparts of AAO and MOX (10). Peroxidases include, among others (69), lignin peroxidase (LiP), manganese peroxidase (MnP), and versatile peroxidase (VP).

Microbial decay of wood and other lignocellulosic materials constitutes an essential step in global geochemical cycles, as it enables the recycling of photosynthetic carbon deposited in the form of structural polymers in plant cell walls (1). The main components of lignocellulosic materials are cellulose, hemicelluloses, and lignin (2, 3). While cellulose and hemicelluloses are sugar polymers, whose amorphous moieties are easily attacked by hydrolytic enzymes, lignin is a highly recalcitrant aromatic polymer which protects plant polysaccharides against microbial and enzymatic attack. Due to its high complexity, decaying fungi have developed an extracellular multienzymatic system to break down the lignin polymer (4–6). This includes lignin peroxidases (LiP), manganese peroxidases (MnP), versatile peroxidases (VP), and laccases, known as ligninolytic enzymes, as well as accessory enzymes, unable to directly degrade lignin, that provide H_2_O_2_ to peroxidases and Fenton reactions, among other roles. These accessory enzymes include glyoxal oxidases (GLOX) and other members of the copper-radical oxidase (CRO) superfamily together, among others, with cellobiose dehydrogenases (CDH), pyranose 2-oxidases (P2O), methanol oxidases (MOX), and aryl-alcohol oxidases (AAO), all of them flavoenzymes belonging to the glucose-methanol-choline oxidase/dehydrogenase (GMC) superfamily (7, 8) (Fig. 1).

AAO activity, that is the release of H_2_O_2_ during aryl alcohol oxidation, was reported for the first time in the 1960s by Farmer et al. (9) (in Trametes versicolor cultures). Since then, a high number of putative AAOs have been identified in basidiomycete and ascomycete genomes and even reported in bacteria and insects, suggesting a widespread presence in nature. All the isolated and characterized AAOs are extracellular flavin adenine dinucleotide (FAD)-containing proteins that catalyze the oxidative dehydrogenation of polyunsaturated alcohols using molecular oxygen (O_2_) as the final electron acceptor and producing H_2_O_2_ (EC 1.1.3.7). However, physiological/biochemical counterparts of AAO and other GMCs, catalyzing similar redox reactions, have also been recently reported in the CRO superfamily (10–12).

In general terms, AAO flavoenzymes are characterized by a broad substrate specificity oxidizing nonphenolic and phenolic aryl alcohols, and even polyunsaturated aliphatic alcohols, with different catalytic efficiencies depending on the enzyme isolation source (13). Among all identified AAOs, the most extensively characterized to date is that from Pleurotus eryngii (PeAAO) (14–25). Its heterologous overexpression in Escherichia coli (26) as well as the information provided by its crystal structure (16) have made possible deeply study of its action mechanism, as a key tool to exploit its catalytic potential, or even redesign it toward the production of bio-based compounds of interest (27–29). PeAAO catalyzes the oxidative dehydrogenation of benzylic alcohols into their corresponding aldehydes through a concerted mechanism for proton abstraction and hydride transfer and the concomitant reduction of O_2_ to H_2_O_2_. This efficient flavooxidase also shows some activity on aldehydes, through their *gem*-diol hydrated forms (17).

Similar AAO flavooxidases, have been isolated from other fungi, including *Pleurotus* and *Bjerkandera* species (30–32). Recently, three aryl-alcohol oxidoreductases were identified in Pycnoporus cinnabarinus (33). These enzymes show high sequence similarity to AAO proteins and even cluster in the same clade of fungal AAO flavooxidases. Although they also share alcohol specificity with AAOs, quinones are their preferential electron acceptors (with residual or insignificant oxidase activity) and were therefore classified as aryl-alcohol dehydrogenases (AADH) (8, 33, 34).

In the present work, we heterologously expressed in E. coli, purified and characterized two AAOs from the genomes of *B. adusta* (BaAAO; JGI ID 171002) and Pleurotus ostreatus (PoAAO; JGI ID 1067653) sequenced at the DOE Joint Genome Institute (JGI; https://mycocosm.jgi.doe.gov) which were compared with the model PeAAO. Besides their physicochemical properties and reducing-substrate specificities, we also analyzed their ability to use *p*-benzoquinone (BQ) as an electron acceptor, in addition to O_2_, a study not addressed before for other AAO flavooxidases. The presence of significant oxidase and dehydrogenase activities in the three studied AAOs suggests additional roles for AAO flavooxidases during plant biomass degradation.

## RESULTS

### E. coli production of *Po*AAO and *Ba*AAO.

E. coli expression of PoAAO and BaAAO led, after 4 h of induction with isopropyl-β-d-thiogalactopyranoside (IPTG), to large quantities of protein accumulated as insoluble inclusion bodies. These inclusion bodies were solubilized in 8 M urea and used to set up the optimal conditions for *in vitro* activation of the two enzymes.

With that aim, several parameters—namely, urea concentration, oxidized/reduced glutathione (GSSG/GSH) ratio, glycerol concentration, and pH—were simultaneously tested in 96-well microplates, and the folding efficiency was followed by *p*-methoxybenzyl alcohol oxidation (as shown in Fig. S1 in the supplemental material for urea and GSSG concentrations). The effect of pH was explored every 0.5 units between pH 7.0 and 9.5, with pH 8.5 and 9.5 being the optima for PoAAO and BaAAO, respectively. No significant increase in refolding efficiency was observed with higher concentrations, and therefore, 20% glycerol was set to facilitate subsequent purification of both AAOs. PoAAO activation was highly influenced by GSSG concentration, achieving optimal folding in the range of 1 to 2 mM GSSG (Fig. S1A), while BaAAO folding was independent of the oxidizing conditions (Fig. S1B). Finally, the urea concentration for an optimal folding was found to be 0.4 M for both AAOs. Regarding temperature, incubation of the folding mixtures at 4°C led to maximal activation after 6 to 7 days.

After refolding, concentration/dialysis, and one anion-exchange chromatographic step (removing unfolded protein and FAD excess), one band was observed in sodium dodecyl sulfate-polyacrylamide gel electrophoresis (SDS-PAGE) for both purified AAOs (Fig. S2). The purification process, summarized in Table 1, allowed recovery of 35 and 21 mg of active PoAAO and BaAAO from 5 L of refolding mixture with specific activities against *p*-methoxybenzyl alcohol of 47 and 24 U·mg^−1^, respectively.

**TABLE 1:** Purification process for recombinant PoAAO and BaAAO after *in vitro* activation from 5 L of refolding mixture

Enzyme	Purification step	Protein (mg)	Activity (U)	Specific act (U/mg)	Purification (fold)	Yield (%)
PoAAO	Refolding mixture	4.2 · 10^3^	4.1 · 10^3^	1.0	1.0	100
	Concent./dialysis	2.1 · 10^2^	1.9 · 10^3^	8.9	9.1	45
	ResourceQ	35	1.6 · 10^3^	47.0	48.0	40
BaAAO	Refolding mixture	4.1 · 10^3^	1.1 · 10^3^	0.3	1.0	100
	Concn/dialysis	96	6.2 · 10^2^	6.4	23.0	54
	ResourceQ	21	5.2 · 10^2^	24.3	87.3	46

The matrix-assisted laser desorption ionization–time of flight (MALDI-TOF) molecular weight (MW) of purified BaAAO was 62,610 Da, and its isoelectric point (pI) from two-dimensional (2D) electrophoresis was 5.6, while PoAAO showed an MW of 60,903 Da and a slightly more acidic pI of 5.0 (Table 2).

**TABLE 2:** Spectroscopic and physicochemical properties of PeAAO, PoAAO, and BaAAO measured at 25°C in 50 mM NaPi, pH 6.0

Type of AAO	Spectroscopic properties			Physical-chemical properties			
	λ_band II_ (nm)	λ_band I_ (nm)	ε_band I_ (M^−1^ cm^−1^)	MW (Da)	pI	*T_m_* (°C)	T_50_ (°C)
PeAAO	386^*a*^	463^*a*^	11,050^*a*^	61,847^*a*^	3.9^*a*^	52.8 ± 0.1^*b*^	—
PoAAO	387	463	9,922	60,903	5.0	50.1 ± 0.1	51.2 ± 0.3
BaAAO	392	463	10,210	62,610	5.6	40.2 ± 0.1	42.8 ± 0.1

a Data from reference 26.

b Calculated in this work.

### Spectral properties.

**FIG 2:**
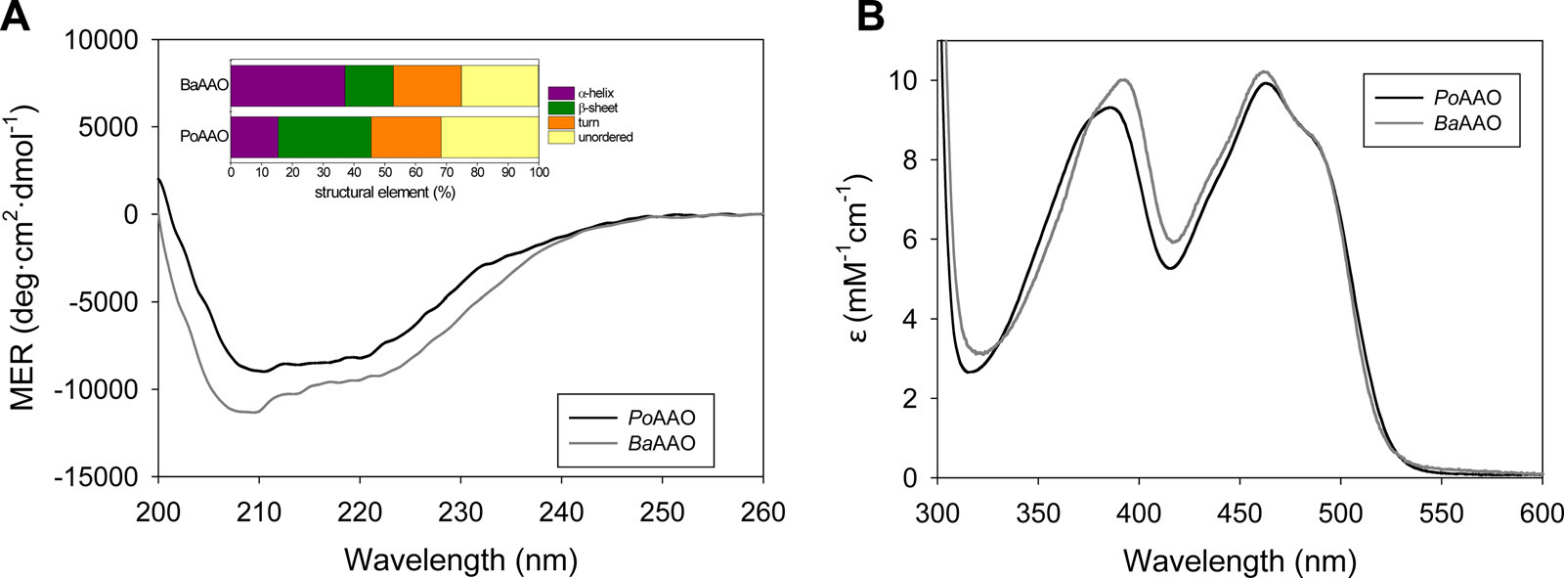
Spectroscopic properties of PoAAO and BaAAO. (A) Far-UV CD spectra; (B) UV-visible spectra of purified PoAAO and BaAAO recorded in 50 mM NaPi, pH 6.0, at 25°C.

The secondary structure of the enzymes was assessed by circular dichroism (CD). Their far UV CD spectra showed a negative-positive couplet at 208 to 200 nm, typical of an α-helix secondary structure, with a shoulder at 222 nm due to the content of β-sheet (Fig. 2A). Further analysis of the CD spectra indicated 15% of α-helix, 30% β-strands, 23% of turns, and 32% of unordered structures for PoAAO, while the content of α-helix increased up to 37% and the content of β-strands decreased to 16% for BaAAO.

Correct incorporation of the cofactor in PoAAO and BaAAO was confirmed by the UV-visible spectra (Fig. 2B and Table 2). Spectra showed the two characteristic peaks of FAD-containing proteins corresponding to flavin band I at 463 nm—with a shoulder at ~490 nm—for both enzymes and band II at 387 and 392 nm for PoAAO and BaAAO, respectively. Moreover, the A_280_/A_463_ ratio was ~10, as expected for flavoenzymes, and their molar absorptivities at 463 nm were 9,922 M^−1^ cm^−1^ and 10,210 M^−1 ^cm^−1^ for PoAAO and BaAAO, respectively.

### Stability of PoAAO and BaAAO.

**FIG 3:**
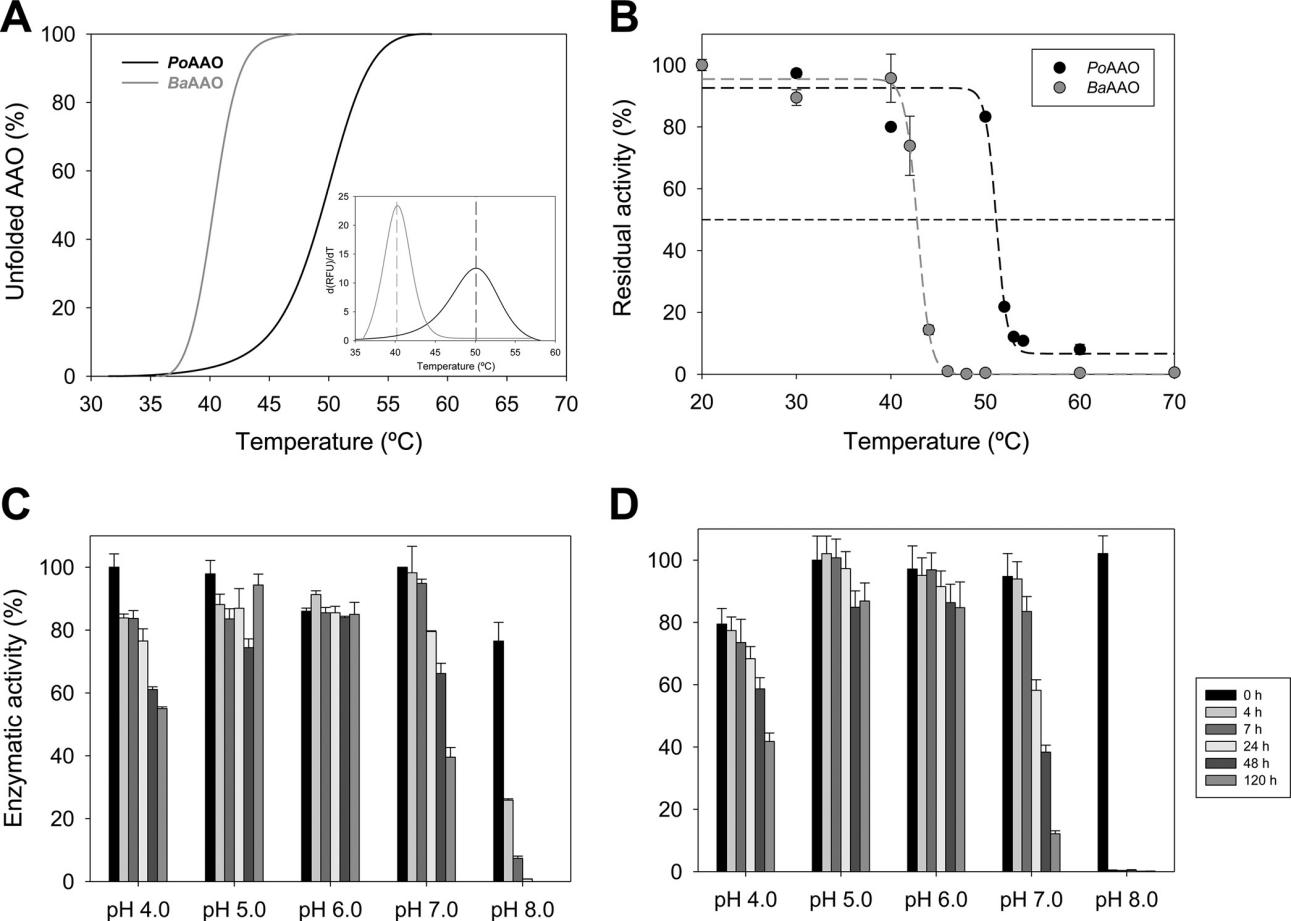
Effect of temperature (top) and pH (bottom) on the stability of PoAAO and BaAAO. (A and B) Thermal stability (A) measured as melting temperature (*T_m_*; PoAAO, black; BaAAO, gray) along a 15 to 90°C temperature ramp (inset show the first derivative of the unfolding curve as a function of temperature) and (B) estimated from residual activity after 10 min of incubation in the range of 20 to 70°C. Assays were performed in 50 mM NaPi, pH 6.0. Lines represent the fittings to a sigmoidal equation. (C and D) pH stability of (C) PoAAO and (D) BaAAO estimated from residual activity immediately after mixing (black bars) and after 4, 7, 24, 48, and 120 h of incubation of enzymes in 100 mM B&R buffer at different pH values (pH 4.0 to 8.0) and 25°C.

The thermal stability of PoAAO and BaAAO was analyzed by both thermal melting (*T_m_*) profiles in denaturation curves and residual activity at different temperatures. The calculated *T_m_* values were 50.2°C and 40.1°C for PoAAO and BaAAO, respectively (Fig. 3A and Table 2), while the temperatures at which they kept 50% of their initial activity (*T*_50_) after a 10-min incubation were 51.3°C and 42.8°C, respectively (Fig. 3B).

The effect of pH on thermal melting profiles for AAOs was first evaluated in the range of pH 3.0 to 10.0, revealing that the enzymes were denaturated below pH 3.0 and above pH 8.5. In the range of pH 3.5 to 8.5 (Fig. S3), PoAAO showed higher *T_m_* values than BaAAO, in line with the thermal stabilities shown above. PoAAO was quite stable in the range of pH 4.0 to 5.5, with a maximal *T_m_* value of 56°C at pH 5.0, while for BaAAO the maximum (48°C) was at pH 5.3. Beyond the above-described pH limits, the enzymes lost stability. Moreover, pH stability over time was analyzed by measuring residual activity during a 5-day incubation in the pH 4.0 to 8.0 range (Fig. 3C and D). After 5 days, both enzymes kept more than 85% of their initial activity at pH 5 to 6, and PoAAO still kept 60% at pH 4 and 40% at pH 7.0 (Fig. 3C), while the BaAAO activity decreased to 40% and 10% (Fig. 3D), respectively.

### Optimal pH and temperature.

**FIG 4:**
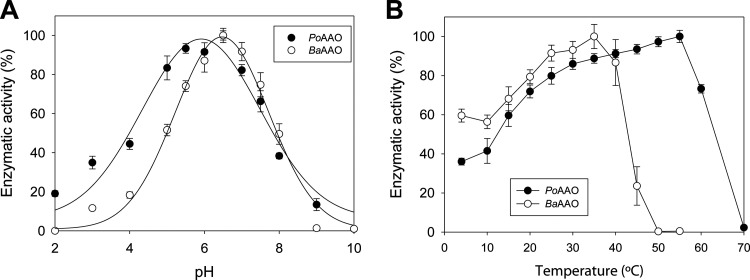
(A and B) Optimal pH (A) and temperature (B) for oxidase activity of PoAAO (closed circles) and BaAAO (open circles). Alcohol oxidase activity was determined using saturating concentrations of *p*-methoxybenzyl alcohol in 100 mM B&R buffer in the pH range of 2.0 to 10.0 at 25°C for the optimal pH assays and in 50 mM NaPi, pH 6.0, at different temperatures (between 4°C and 60°C) for the optimal temperature assays.

The optimal pH (Fig. 4A) was 6.0 for PoAAO, with more than 70% of activity in the pH 4.5 to 7.0 range, and 6.5 for BaAAO, with a slightly narrower range. No activity was detected either below pH 3.0 or over pH 9.0 for both enzymes.

The optimal temperature, with more than 80% activity, was in the ranges of 25 to 55°C for PoAAO and 20 to 40°C for BaAAO (Fig. 4B). Moreover, the BaAAO activity decreased to 30% at 45°C with complete loss of activity above 50°C, while PoAAO kept 60% activity at 60°C, and was completely inactivated only at 70°C.

### Kinetic properties of PoAAO and BaAAO.

The steady-state curves and resulting kinetic parameters for oxidation of different alcohols under atmospheric conditions with O_2_ as an electron acceptor were compared for PoAAO, BaAAO, and PeAAO (Fig. S4 and Table S1). The three enzymes oxidized the whole spectrum of alcohols although with different catalytic efficiencies. The BaAAO produced in E. coli showed kinetic constants similar to those reported for the wild (i.e., nonrecombinant) AAO from *B. adusta* (32), with the highest catalytic efficiency for 3-chloro-*p*-methoxybenzyl alcohol, followed by 3-chlorobenzyl and *p*-methoxybenzyl alcohols, while the best substrate for PoAAO and PeAAO was *p*-methoxybenzyl alcohol, followed by 3-chloro-*p*-methoxybenzyl alcohol and 2,4-hexadien-1-ol (with *k*_cat_/*K_m_* values of approximately 18-, 5-, and 30-fold higher, respectively, than those of BaAAO).

### Use of different electron acceptors.

The ability of PeAAO, PoAAO, and BaAAO to use electron acceptors other from O_2_ was first tested under anaerobic conditions in 96-well plates with *p*-methoxybenzyl alcohol and 2,6-dichlorophenolindophenol (DCPIP) as the electron donor and acceptor, respectively. The observed *v*_0_/[AAO] values (initial reaction velocity per μmol AAO) were independent of DCPIP concentrations (100 to 400 μM), suggesting saturation at the concentrations assayed. The three enzymes reduced DCPIP although with different rates (Fig. S5). Under the experimental conditions used, PoAAO was the fastest for DCPIP reduction with *v*_0_/[AAO] values 2.5- and 1.6-fold higher than for PeAAO and BaAAO, respectively.

In the light of these results, bi-substrate kinetics (varying both oxidizing and reducing substrate concentrations) were performed for oxidation of *p*-methoxybenzyl alcohol with either O_2_ or BQ as the electron acceptor. In all the cases, double reciprocal plots (*e*/*v*_0_ versus 1/[*p*-methoxybenzyl alcohol]) gave parallel lines, indicating that the reactions follow a ping-pong mechanism (Fig. S6). This mechanism was confirmed by the linear patterns obtained in double reciprocal plots of *e*/*v*_0_ versus 1/[*p*-methoxybenzyl alcohol] when the concentrations of the two substrates were kept at a fixed ratio (Fig. S7).

Thus, experimental data were fitted to equation 3, below, to obtain the kinetic parameters (for *p*-methoxybenzyl alcohol oxidation) shown in Table 3. In general, PeAAO and PoAAO showed similar turnover numbers (*k*_cat_) and *K_m_* values when using the same electron acceptor, either O_2_ or BQ, while BaAAO showed lower activity in both O_2_ (2- to 3-fold lower *k*_cat_) and BQ (1.5- to 1.8-fold lower *k*_cat_) reactions. Moreover, the three enzymes showed higher *k*_cat_ when O_2_ was the electron acceptor (4.1-, 2.8-, and 2.4-fold higher for PeAAO, PoAAO, and BaAAO, respectively). In contrast, all enzymes showed a lower *K_m_* (i) for BQ than for O_2_ and (ii) for the alcohol substrate in the presence of BQ. This results in similar, or higher, catalytic efficiencies when BQ was used as the electron acceptor compared to O_2_.

**TABLE 3:** Bi-substrate steady-state kinetic parameters for the oxidation of *p*-methoxybenzyl alcohol by PeAAO, PoAAO and BaAAO at 25°C with O_2_ or BQ as electron acceptors in 50 mM NaPi, pH 6.0, obtained from equation 3

Enzyme	Acceptor	*k*_cat_ (s^−1^)	*K_m_*_(acceptor)_ (μM)	*K_m_*_(alcohol)_ (μM)	*k*_cat_/*K_m_*_(acceptor)_ (mM^−1^ s^−1^)	*k*_cat_/*K_m_*_(alcohol)_ (mM^−1^ s^−1^)
PeAAO	O_2_	201 ± 3	176 ± 5	50 ± 2	1,140 ± 40	4020 ± 140
	BQ	49 ± 4	48 ± 6	7 ± 1	1,030 ± 150	6,590 ± 960
PoAAO	O_2_	156 ± 4	144 ± 8	43 ± 3	1,080 ± 70	3,610 ± 240
	BQ	56 ± 3	33 ± 4	13 ± 1	1700 ± 210	4,340 ± 470
BaAAO	O_2_	74 ± 1	134 ± 5	331 ± 13	550 ± 20	220 ± 10
	BQ	31 ± 3	70 ± 9	82 ± 10	450 ± 70	380 ± 60

**FIG 5:**
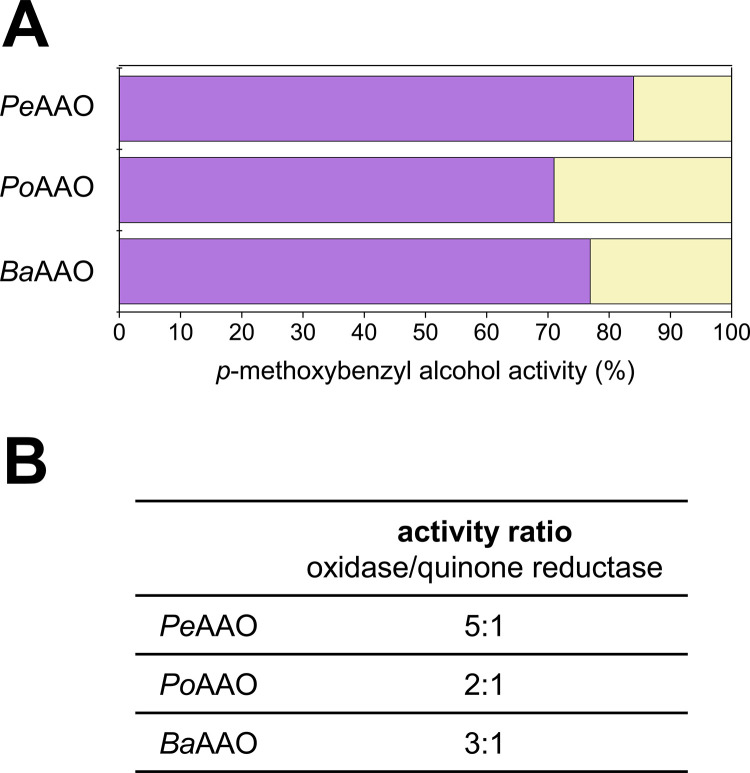
Relative contribution of oxidase and quinone reductase activities of AAOs to alcohol oxidation. (A) Oxidase (purple) and quinone reductase (yellow) activities (%) estimated for the oxidation of saturating concentration of 1.5 mM *p*-methoxybenzyl alcohol in the presence of 120 μM BQ in air-saturated (256 μM O_2_) 50 mM NaPi, pH 6.0, at 25°C (see Materials and Methods for details of activities estimation). (B) Different activity ratios in the PeAAO, PoAAO, and BaAAO reactions.

These unexpected results led us to investigate if under atmospheric conditions quinone reductase activity could be detectable in AAO reactions. Thus, the contributions of oxidase and quinone reductase to oxidation of 1.5 mM *p*-methoxybenzyl alcohol in air-saturated 50 mM NaPi, pH 6.0, were estimated as detailed in Material and Methods. In the presence of both electron acceptors, the alcohol oxidation by O_2_ was more favorable, probably due to the free diffusion of this acceptor to the active site. In addition, the entrance of BQ, or the release of *p*-hydroquinone, through the active-site channel might be hampered, as reported for aldehyde product release in PeAAO (24). Either way, operation of the AAO quinone reductase activity was demonstrated in the presence of O_2_, as shown in the Fig. 5 experiments carried out under atmospheric conditions.

### Structural modeling of PoAAO and BaAAO.

PoAAO and BaAAO sequences were modeled using the AlphaFold server, and conservation of their amino acid sequences was evaluated using the ConSurf server (Fig. S8B and C). Similar to the PeAAO crystal structure (Fig. S8A), the models for PoAAO and BaAAO predicted a fold into the two domains characteristic of the GMC superfamily: a lower domain harboring the FAD cofactor and an upper substrate-binding domain, the latter being less conserved. The molecular models and secondary structure distribution in the three above-described AAOs were compared with similar models of the three AADHs from *P. cinnabarinus* (Fig. S8D to F).

The putative active sites in PoAAO and BaAAO are located at the *re* face of the cofactor’s isoalloxazine ring and include two strictly conserved histidine residues (H506/H550 and H513/H555 for PoAAO and BaAAO, respectively) that play a decisive role in substrate oxidation by PeAAO (19). As described for PeAAO (Fig. S9A) (16), the entrance to the putative active site in both AAO models is through a narrow channel reaching the isoalloxazine ring and predicted to be highly variable in sequence (Fig. S9B and C). Moreover, the channel of PoAAO presents a highly hydrophobic funnel-like bottleneck (Y95, F401, and F505) similar to those residues identified in PeAAO, while a wider channel, limited by F104, T408, and W512, is predicted in BaAAO (Fig. S9A to C). The active-site access channels were also predicted in the AADH models (Fig. S9D to F).

## DISCUSSION

### Two new recombinant AAOs.

AAOs from *B. adusta* and *P. ostreatus*, which respectively share 46.9% and 98.1% sequence identity with *P. eryngii* AAO, were heterologously expressed as E. coli inclusion bodies, *in vitro* activated, purified, and characterized. During activation, the oxidizing conditions provided by GSSG favor formation of the disulfide bridge expected between the two cysteine residues (C252 and C267) of PoAAO, while for BaAAO, with only one cysteine, oxidizing conditions are not required. In PeAAO, two equivalent cysteines (C248 and C263) form a disulfide bridge at the FAD-binding domain (16). The lack of such an interaction in BaAAO could explain its lower stability (*T_m_* and *T*_50_) compared to the *Pleurotus* AAOs (Table 2). Moreover, the lower stability of recombinant BaAAO compared to the fungal enzyme (32) can be attributed to the lack of glycosylation, as previously reported for PeAAO (26).

### Different AAO electron donors and acceptors.

**FIG 6:**
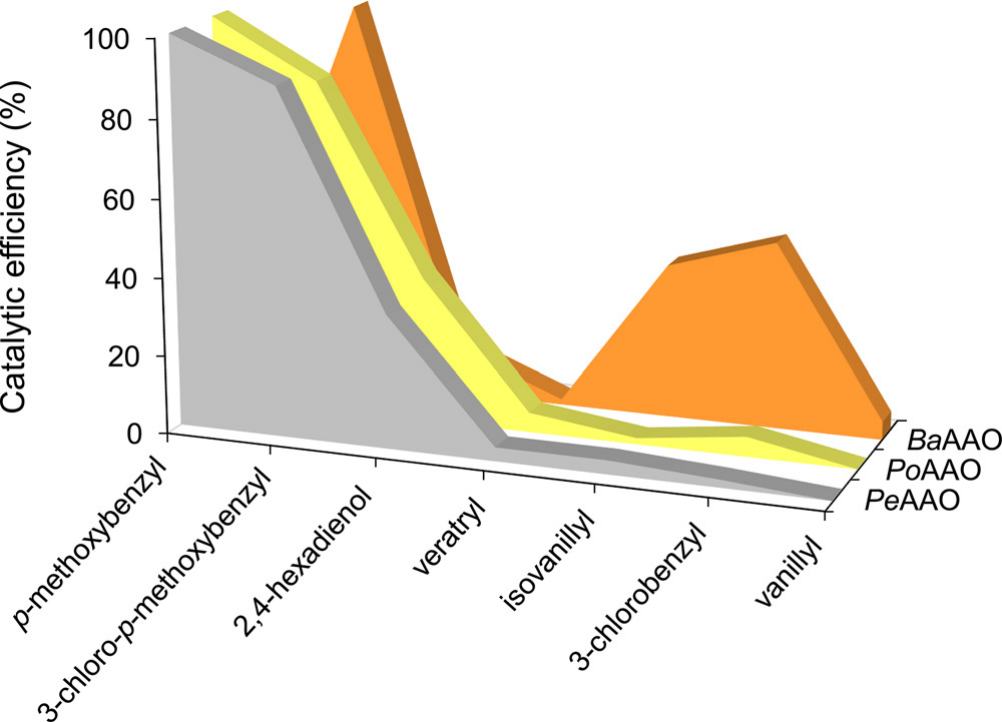
Catalytic efficiency of AAOs on different alcohol substrates. Relative catalytic efficiencies (*k*_cat_/*K_m_* values from Table S1) of PeAAO (gray), PoAAO (yellow), and BaAAO (orange) on different alcohol substrates, with the maximal value for each enzyme taken as 100%. Kinetics were performed at 25°C in air-saturated 50 mM NaPi, pH 6.0.

The kinetic parameters for oxidation of alcohol substrates revealed differences between the new PoAAO and BaAAO. The former enzyme showed a substrate spectrum similar to that of the model *P. eryngii* enzyme, displaying the highest catalytic efficiency for the oxidation of *p*-methoxybenzyl alcohol (Fig. 6). In contrast, the substrate spectrum (in terms of catalytic efficiency) of BaAAO was different, with higher values for oxidation of the chlorinated 3-chloro-*p*-methoxybenzyl and 3-chlorobenzyl alcohols. This difference in substrate specificity, already reported for the wild BaAAO (32), reveals that each AAO is more efficient at oxidizing those benzylic alcohols that are more abundant in its natural environment. Note that *p*-methoxybenzyl alcohol is a typical metabolite in *Pleurotus* species (35, 36), while its chlorinated counterparts are synthesized by *Bjerkandera* species (37, 38).

Concerning electron acceptors, our results show how both O_2_ and BQ can be natural oxidizing substrates of AAOs. BQ and/or phenoxy radical reduction by GMC flavooxidases was suggested years ago in the context of lignin biodegradation (39–41). The results obtained here illustrate how both activities can compete when the two electron acceptors, O_2_ and BQ, are simultaneously present. The quinone-reductase/oxidase maximal turnover ratios of PeAAO, PoAAO, and BaAAO were 1:4, 1:3, and 1:2, respectively (Table 3). These values contrast with the 50:1 ratios reported for two of the AADHs of *P. cinnabarinus* (PcAADH2 and PcAADH3) and PcAADH1’s complete lack of oxidase activity (33), in agreement with the dehydrogenase nature of the three latter GMCs. Interestingly, the catalytic efficiencies of the three AAOs with BQ as an electron acceptor are substantially higher than those reported for the *P. cinnabarinus* AADHs. These results show that AAOs are not only efficient alcohol oxidases but can also be efficient alcohol dehydrogenases, using BQ as electron acceptor. The above-described findings were confirmed in alcohol oxidation experiments under atmospheric conditions, where 16%, 29%, and 23% contributions of quinone reductase activity were estimated for PeAAO, PoAAO, and BaAAO, respectively. These results, together with the comparatively low *K_m_* for BQ, indicate that both activities—oxidase and dehydrogenase—would compete in nature depending on the availability of quinones and O_2_ in the enzyme microenvironment. All these data suggest a biological role for the quinone reductase activity of AAOs, preventing radical repolymerizations, as claimed also for one of the best known quinone-reducing enzymes in white-rot fungi (CDH) (42), together with other eventual roles of AAO discussed below.

The residual or even absent reactivity of AADH enzymes with oxygen has been related with a wider access channel to the active site and subtle amino acid changes along this channel (8, 33). Our structural models predicted a similar overall folding for both AAOs and AADHs, as expected for members of the GMC oxidoreductase superfamily (43), with highly conserved residues in the FAD-binding domain, while more variability is predicted in the substrate-binding domain, as shown by the ConSurf conservation scores (Fig. S8). A closer look into the active-site access showed that residues forming the channel are highly variable, as shown by the blue color assigned by ConSurf (Fig. S9). In *Pe*AAO/*Po*AAO, three aromatic residues—Y92/Y95, F501/F505, and F397/F401—form a bottleneck that restricts the access of substrates to the active site. In the other enzymes, despite showing different residues at these positions, the hydrophobicity is maintained, except for BaAAO (in which T408 replaces F397) and PcAADH1 (in which R514 replaces F501). In PcAADH2, despite keeping the hydrophobicity, two of the aromatic residues are replaced by L103 and A407, and in PcAADH3 two tryptophan residues (W104 and W517) accompany F413 at the access channel. These subtle changes are not sufficient to explain the absence/presence of oxidase and dehydrogenase activity in AAOs and AADHs, and further studies are therefore required.

In PeAAO, the diffusion of oxygen to the active site has been related to its oxidase efficiency, with F501 and F397 residues helping oxygen to attain its catalytically competent position near the flavin C4a and catalytic histidine (18, 24). Moreover, alcohol substrate migration to AAO active site takes place through the same channel, requiring important reorganization of the F397 and Y92 side chains. Therefore, a similar pathway and requirements for the quinone entrance to the AAO active site are expected in these enzymes. In fact, mutation of both the active site and its access channel has been shown to modulate the electron-acceptor preference of flavin-containing oxidases and dehydrogenases (44) for biosensor application (45).

### Biological significance of quinone reductase activity.

**FIG 7:**
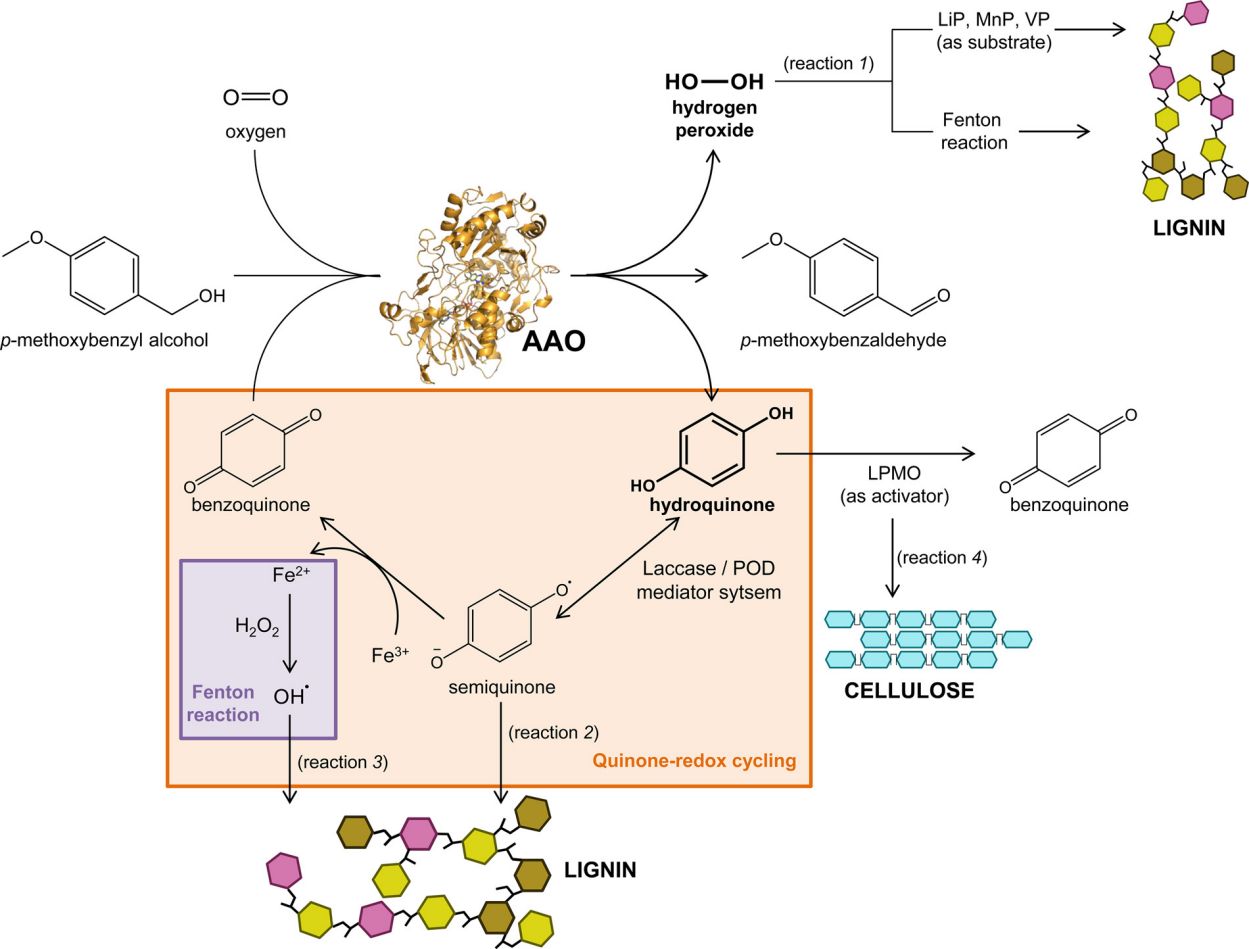
Scheme of the proposed roles of AAO in lignocellulose degradation. AAO as an oxidase participates in lignin attack generating the H_2_O_2_ required for ligninolytic peroxidases and Fenton reactions (reaction 1). Additionally, the AAO ability to reduce BQ to hydroquinone expands its potential by providing laccase and POD mediator systems (reaction 2), generating reactive hydroxyl radicals (reaction 3), and fueling the LPMO activity involved in crystalline cellulose degradation (reaction 4).

So far, the main role assigned to AAOs in lignocellulosic decay is the continuous supply of H_2_O_2_ required as a cosubstrate of ligninolytic peroxidases or to trigger Fenton reactions (46) (Fig. 7, reaction 1). Although the eventual effect of the presence of glycosidic moieties in the wild enzymes cannot be discarded (substrate diffusion to the active site, product release, or others) (47, 48), the ability to reduce BQ demonstrated here for the E. coli-expressed AAOs suggests several potential additional roles of the wild fungal enzymes during biomass decay.

On the one hand, in addition to preventing the repolymerization of semiquinones and other lignin radicals, as mentioned above, the resulting hydroquinones/phenols can act as natural redox mediators (49, 50) of laccases (and low-redox potential peroxidases) in the oxidative degradation of lignin. Hydroquinones are one-electron oxidized to stable semiquinone radicals that can serve as electron shuttles in the oxidation of lignin polymers (Fig. 7, reaction 2). Moreover, semiquinones, being able to reduce ferric iron, drive the production of reactive hydroxyl radicals through a Fenton reagent (Fe^2+^ + H_2_O_2_) (51). These radicals can attack lignin to facilitate the action of lignocellulolytic enzymes (Fig. 7, reaction 3). Thus, the formation of hydroquinone by AAO when using BQ as an electron acceptor could trigger the quinone-redox cycling, as described in white-rot fungi.

On the other hand, focusing on cellulose degradation, an efficient electron-shuttle system for the activation of lytic polysaccharide monooxygenases (LPMO) has been reported in which hydroquinones act as redox mediators between LPMO and GMC oxidoreductases, mainly dehydrogenases but also including glucose oxidase, able to reduce BQs (52, 53). In the same way, the new quinone reductase activity reported here for AAO could fuel the action of LPMO for the initial oxidative attack to recalcitrant crystalline cellulose (Fig. 7, reaction 4).

**(i) Concluding remarks.** The two recombinant AAOs characterized in this work (i.e., PoAAO and BaAAO), as well as the model PeAAO, have different substrate specificities and catalytic efficiencies correlating with the alcohol availability for each fungal species in nature. More interestingly, the ability of the three AAOs to efficiently use BQ as an electron acceptor suggests roles other than those previously assigned. In this way, AAO (i) acts as an auxiliary enzyme for ligninolytic peroxidases by supplying H_2_O_2_, (ii) would prevent repolymerization of semiquinone and other lignin radicals, (iii) can be an auxiliary enzyme for the action of LPMO, (iv) can fuel the action of laccases providing reduced quinones as redox mediators, and (v) can also fuel the production of reactive hydroxyl radicals through a Fenton reaction.

## MATERIALS AND METHODS

### Chemicals.

*p*-Methoxybenzyl, 3-chlorobenzyl, 4-hydroxy-3-methoxybenzyl (vanillyl), 3,4-dimethoxybenzyl (veratryl) and 3-hydroxy-4-methoxybenzyl (isovanillyl) alcohols, 2,4-hexadien-1-ol, and *p*-benzoquinone were obtained from Sigma-Aldrich (St. Louis, MO, USA). AmplexRed and horseradish peroxidase were purchased from Invitrogen (Waltham, MA, USA). 3-Chloro-*p*-anisyl alcohol was synthesized at the Instituto de Nanociencia y Ciencia de Materiales de Aragón (CSIC-UZ, Zaragoza, Spain). DCPIP was obtained from Merck.

### AAO production in E. coli and *in vitro* activation.

Recombinant AAO from *P. eryngii* was obtained by E. coli expression of the mature AAO cDNA (GenBank AF064069) followed by *in vitro* activation in the presence of the cofactor and purification by ion-exchange chromatography as previously described (26).

Codifying DNA sequences of AAO from *P. ostreatus* and *B. adusta*, excluding signal peptide identified with SignalP3.0, were taken from the corresponding fungal genomes sequenced at JGI (JGI ID 1067653 and ID 171002, respectively), manually optimized for E. coli expression, and synthesized by ATG:biosynthetics. These sequences were subcloned from the pGH vector into the NdeI/BamHI restrictions sites of pET23b(+). For production, E. coli C41(DE3) cells—transformed with the corresponding expression vectors—were grown overnight at 37°C with continuous shaking at 180 rpm in lysogeny broth (LB) medium supplemented with 100 μg/mL of ampicillin. The precultured cells were used to inoculate 1 L of LB (supplemented with ampicillin) that was incubated at 37°C under continuous shaking at 180 rpm until reading an optical density of 500 nm (OD_500nm_) of ~0.9. Cultures were induced with 1 mM IPTG, grown for another 4 h, and then harvested by centrifugation. Protein expression was monitored using 12% SDS-PAGE.

The bacterial pellets were resuspended in lysis buffer, 50 mM Tris/HCl, pH 8.0, containing 10 mM ethylenediaminetetraacetic acid (EDTA), and 5 mM dithiothreitol (DTT) and incubated with 2 mg/mL lysozyme and 0.1 mg/mL DNase I for 45 min on ice. Then, the solutions were sonicated and centrifuged. Pellets containing the enzymes as inclusion bodies were washed with 1 mM EDTA and 5 mM DTT in 20 mM Tris/HCl, pH 8.0. The protein was solubilized with 8 M urea, 1 mM EDTA and 5 mM DTT in 50 mM Tris/HCl, pH 8.0, and then centrifuged to eliminate insoluble debris. Protein concentration was determined with the Bradford method using the protein assay (Bio-Rad) and bovine serum albumin as the standard.

The optimal conditions for *in vitro* activation of AAOs were determined in a 96-well-plate screening (Fig. S1) by mixing the refolding mixture with various concentrations of urea (0.1 to 1.2 mM), oxidized glutathione (GSSG) (0.1 to 3 mM), and glycerol (20% and 40%) in 20 mM Tris/HCl in the pH range of 7.0 to 9.5 and incubation at 4°C. Enzyme (0.15 mg/mL), FAD (0.02mM), and DTT (1 mM) concentrations remained constant in the screening assay. Refolding efficiency was estimated from *p*-methoxybenzyl alcohol oxidation in the above-described mixtures.

The refolding mixture was 25-fold concentrated by tangential ultrafiltration with a 10-kDa membrane (Pellicon, Millipore). After successive concentration steps, first with sucrose and then with a stirred ultrafiltration pressure-based cell with a 10-kDa membrane (Amicon, Millipore), the active enzymes were purified by anionic exchange chromatography, using a ResourceQ column (GE Healthcare) with a 0 to 300 mM NaCl gradient in 50 mM NaPi, pH 6.0. Samples were dialyzed in 50 mM NaPi, pH 6.0, and conserved at −80°C.

### Spectroscopic properties.

UV-visible spectra of the purified enzymes were recorded between 250 and 700 nm in a Cary4000 spectrophotometer. The extinction coefficients of PoAAO and BaAAO were determined upon release of FAD (54) after thermal denaturation (5 min at 100°C) (26).

Far-UV CD spectra of AAOs (5 μM) were recorded in a Jasco J-815 spectropolarimeter at 25°C in 5 mM NaPi, pH 6.0, in a 0.1-cm-path-length cuvette. The spectra were analyzed with the CDPro programs SELCON3 (55), CDSSTR (56), and CONTINLL (57) to determine the secondary structure.

### MW and pI determination.

The MW analyses by mass spectrometry (MS)-MALDI TOF were carried out at the Proteomics and Genomics Facility (of CIB-CSIC), a member of the ProteoRed-ISCIII network. The experiments were performed on an Autoflex III MALDI-TOF-TOF instrument (Bruker Daltonics, Bremen, Germany) with a Smartbeam laser. Samples dissolved in 50 mM NaPi, pH 6.0, were mixed with 2% trifluoroacetic acid and with the 2,5-dihydroxyacetophenone matrix solution (dissolved in 75% ethanol 80 mM diammonioum hydrogen citrate). External calibration was performed using the bovine albumin from Sigma, covering a range of 20,000 to 100,000 Da.

The pI was determined by 2D electrophoresis. The first dimension was run on immobilized pH gradient strips (pH 3 to 10 linear, 7 cm) (Bio-Rad), and the second dimension was run on 12% SDS-PAGE. Protein spots were stained with a colloidal blue staining kit (Invitrogen).

### Thermal stability.

Two methods were used to evaluate the thermal stability of the enzymes. First, the *T_m_* of the enzymes (20 μM) were analyzed using the ThermoFAD method (58). The increase in fluorescence due to FAD released during enzyme unfolding was monitored with a real-time PCR thermocycler iQ5 (Bio-Rad). *T_m_* values were obtained from the maximum of the first derivative of the unfolding curve.

Second, the enzymes (~6 μM) were incubated in 50 mM NaPi, pH 6.0, at different temperatures (from 20 to 80°C). After 10 min, they were allowed to rest for 2 min at 4°C, and the remaining activities were measured as described above. The *T*_50_ value, defined as the temperature at which the enzyme keeps 50% of activity after a 10-min incubation, was calculated by fitting the data to a sigmoidal equation.

### pH stability.

The pH stability was estimated using thermal melting profiles of ~20 μM enzyme in 100 mM Britton & Robinson buffer in the range of pH 2.0 to 10.0 with the ThermoFAD method, mentioned above. pH stability along time was measured by incubating the purified enzymes (~6 μM) in 100 mM Britton & Robinson (B&R) buffer at different pH values (pH 4.0 to 8.0). Residual activities were estimated by following the oxidation of saturating concentrations of *p*-methoxybenzyl alcohol (180 μM for PeAAO and PoAAO and 500 μM for BaAAO) in 50 mM NaPi, pH 6.0, at 25°C, immediately after mixing and after 4, 7, 24, 48, and 120 h of incubation at 25°C. The highest activity after mixing (at any pH) was taken as 100% activity, and the percentages of residual activity at the different times and pH conditions were calculated according to this maximal value. In both cases, experimental data were fitted to a Gaussian equation to determine the maximal pH value for each enzyme.

### Optimal pH and temperature for oxidase activity.

The optimal pH values for *p*-methoxybenzyl oxidation were determined by measuring the oxidation of saturating substrate concentrations (180 μM for PeAAO and PoAAO and 500 μM for BaAAO) in 100 mM B&R buffer in the range of pH 2.0 to 10.0, as described above.

The optimal temperature was determined by measuring the oxidation of *p*-methoxybenzyl alcohol (180 μM for PeAAO and PoAAO, and 500 μM for BaAAO) in 50 mM NaPi, pH 6.0, at different temperatures, between 4°C and 60°C.

### Microtiter plate activity assay.

Reduction of DCPIP was analyzed in 96-well plates using a SpectraMax Plus plate reader (Molecular Devices, California, USA) in kinetic mode. Each well contained 100 μL of the assay mixture, which was composed of the electron acceptor (100, 200, 300, and 400 μM DCPIP concentrations) and *p*-methoxybenzyl alcohol substrate (1 mM) in 50 mM NaPi, pH 6.0. Mixtures were prepared in an anaerobic chamber to achieve anaerobic conditions, and the reaction was initiated by the addition of 100 nM enzyme. DCPIP reduction was followed at 25°C by changes in absorbance at 600 nm. The DCPIP concentration was calculated using an optical path length of 0.354 cm and an extinction coefficient for DCPIP of 19,100 M^−1^ cm^−1^ (calculated for these experimental conditions). Initial rates were fitted to a linear equation to obtain *k*_obs_ (where obs stands for observed rate constant) as μmol of DPIP reduced per μmol of enzyme per min.

### Steady-state kinetics measurements.

Steady-state kinetic parameters were measured spectrophotometrically by monitoring the oxidation of the different alcohols to the corresponding aldehydes—Δε_285_, 16,950 M^−1^ cm^−1^ for *p*-methoxybenzyl alcohol; Δε_240_, 5,923 M^−1^ cm^−1^ for 3-chloro-benzyl alcohol; Δε_309_, 8,332 M^−1^ cm^−1^ for vanillyl alcohol; Δε_307_, 7,383 M^−1^ cm^−1^ for isovanillyl alcohol; Δε_280_, 30,140 M^−1^ cm^−1^ for 2,4-hexadien-1-ol from (15); Δε_295_, 15,000 M^−1^ cm^−1^ for 3-chloro-*p*-mehxoybenzyl alcohol from (38); and Δε_310_, 9,300 M^−1^ cm^−1^ for veratryl alcohol from Guillén et al. (59)—in air-saturated (0.256 mM O_2_ concentration) 50 mM NaPi, pH 6.0, at 25°C.

Bi-substrate kinetics (60) were performed by simultaneously varying the concentration of *p*-methoxybenzyl alcohol (1 to 125 μM for all the BQ reactions and 5 to 180 μM for PeAAO and PoAAO and 40 to 1,000 μM for the BaAAO reactions with O_2_) and electron acceptor (6 to– 68 μM BQ and 48 to 1,220 μM O_2_) in 50 mM NaPi, pH 6.0, at 25°C. Reactions with O_2_ as the electron acceptor were carried out in a screw-cap cuvette where the alcohol was equilibrated with the desired concentration of O_2_ by bubbling for 10 min with the appropriate O_2_/N_2_ gas mixture (4%, 10%, 21%, 44%, and 100% O_2_, corresponding to 48, 122, 256, 537, and 1220 μM O_2_, respectively, in solution at 25°C). The enzymatic reaction was initiated by the addition of the enzyme (~5 nM for PeAAO and PoAAO and ~20 nM for BaAAO) and was followed at 285 nm. When anaerobic conditions were required, O_2_ was removed by bubbling the samples containing the alcohol substrate with a mixture of CO_2_ + N_2_ for 10 min. Kinetics were monitored following the reduction of BQ at 247 nm. The corresponding extinction coefficient (Δε_247_, 17,833 M^−1^ cm^−1^) was previously calculated taking into account the Δε_247_ value of 20,200 M^−1^ cm^−1^ for the reduction of BQ to hydroquinone (61) and the contribution of *p*-methoxybenzyl alcohol oxidation at this wavelength.

Steady-state kinetic analyses and statistical fits were performed using the SigmaPlot program. Apparent kinetic parameters in atmospheric oxygen were determined by fitting initial reaction rates (*v*_0_) at different alcohol concentrations to the Michaelis-Menten equation (equation 1).

(1) $$\frac{v_{0}}{e}=\frac{k_{\text{cat}}[A]}{K_{m}+[A]}$$

Initial rates for bi-substrate kinetics were fit to equations 2 and 3, which describe sequential and ping-pong kinetic mechanisms, respectively:

(2) $$\frac{v_{0}}{e}=\frac{k_{\text{cat}}AB}{K_{m}^{B}A+K_{m}^{A}B+\mathrm{AB}+K_{d}^{A}K_{m}^{B}}$$

(3) $$\frac{v_{0}}{e}=\frac{k_{\mathrm{cat}}AB}{K_{m}^{B}A+K_{m}^{A}B+\mathrm{AB}}$$

where *v*_0_ is the initial velocity, *e* represents the enzyme concentrations, *k*_cat_ is the maximum turnover (catalytic constant), *A* is the alcohol concentration, *B* is the electron acceptor concentration, $K_{m}^{A}$ and $K_{m}^{B}$ are the Michaelis constants for *A* and *B,* respectively, and $K_{d}^{A}$ is the dissociation constant for *A*.

When the ratio of the concentrations of alcohol and electron acceptor was kept equal to a constant value α (i.e., *A*/*B* = α), equations 4 and 5 were used. In this case, double reciprocal representation gives a straight line for a ping-pong mechanism and a polynomial curve for a sequential mechanism with the formation of a ternary complex (62, 63).

(4) $$\frac{e}{v_{0}}=\left[(K_{m}^{A}\;+\;\alpha K_{m}^{B})\frac{1}{A}+1\right]\left(\frac{1}{k_{\text{cat}}}\right)$$

(5) $$\frac{e}{v_{0}}=\left[\alpha K_{d}K_{m}^{B}\frac{1}{A^{2}}\;+(K_{m}^{A}+\alpha K_{m}^{B})\frac{1}{A}+1\right]\left(\frac{1}{k_{\text{cat}}}\right)$$

Reactions in the presence of both electron acceptors were carried out in air-saturated (256 μM O_2_) 50 mM NaPi, pH 6.0, at 25°C with saturating concentrations of *p*-methoxybenzyl alcohol (~1.5 mM) and BQ (~120 μM). Alcohol oxidation was initiated by the addition of the enzyme (5 nM for PeAAO and PoAAO and 50 nM for BaAAO). The oxidase activity was determined by following the production of H_2_O_2_ using an horseradish peroxidase (HRP)-coupled assay with AmplexRed (Δε_563_, 52,000 M^−1^ cm^−1^) as the final substrate. The reduction of BQ was followed at 247 nm to determine the quinone reductase activity. The contribution of oxidase and quinone reductase activities under these conditions was calculated taking the sum of both activities as 100%.

### Bioinformatic tools.

The AlphaFold sever (64, 65) was used to build the structural models of PoAAO, BaAAO, and AADH from *Pycnoporus cinnabarinus* (GenBank protein number ALS87661 for AADH1, ALS87662 for AADH2, and ALS87663 for AADH3). Conservation scores were calculated with the ConSurf server (66) using only the protein sequence of interest as input. Active-site access channels in protein structures were calculated with the CAVER 3.0.3 PyMOL plugin (67), setting as starting point the center among N5(FAD), Nε2(H501), and Nε2(H546). The PyMOL Molecular Graphics System was used to produce the structural representations and to visualize predicted channels (68).
