# Supplementary material for: Expanding the Physiological Role of Aryl-Alcohol Flavooxidases as Quinone Reductases
Source: Appl Environ Microbiol. 2023 May 8;89(5):e01844-22. doi: 10.1128/aem.01844-22 (PMC10231232; doi:10.1128/aem.01844-22)
Supplement: Supplemental file 1 — Supplemental material. Download aem.01844-22-s0001.pdf, PDF file, 1.7 MB [file aem.01844-22-s0001.pdf]

Supplementary information

Expanding the physiological role of aryl-alcohol flavooxidases as quinone reductases

Patricia Ferreira,[a] Juan Carro,[b] Beatriz Balcells,[b] Angel T. Martínez,[b#] and Ana Serrano[b†#]

[a] Facultad de Ciencias and Instituto de Biocomputación y Física de Sistemas Complejos, Zaragoza, Spain
[b] Centro de Investigaciones Biológicas "Margarita Salas", CSIC, Madrid, Spain

[†]Current address: Certest Biotec SL, San Mateo de Gállego, Zaragoza, Spain
[#]Corresponding authors: anaserra1979@gmail.com and atmartinez@cib.csic.es

Supplementary information includes: *In vitro* activation of AAOs (**Figure S1**), Purification of AAOs (**Figure S2**), pH stability of AAOs (**Figure S3**), Steady-state kinetics for oxidation of different substrates by AAOs (**Figure S4**), DCPIP reduction (**Figure S5**), Bi-substrate kinetics for AAOs (**Figure S6**), Double reciprocal plots (**Figure S7**), Overall secondary structure for AAOs and AADHs (**Figure S8**), Detail of the active sites and access channel to the active site of AAOs and AADHs (**Figure S9**) and Steady-state kinetic parameters of AAOs (**Table S1**).

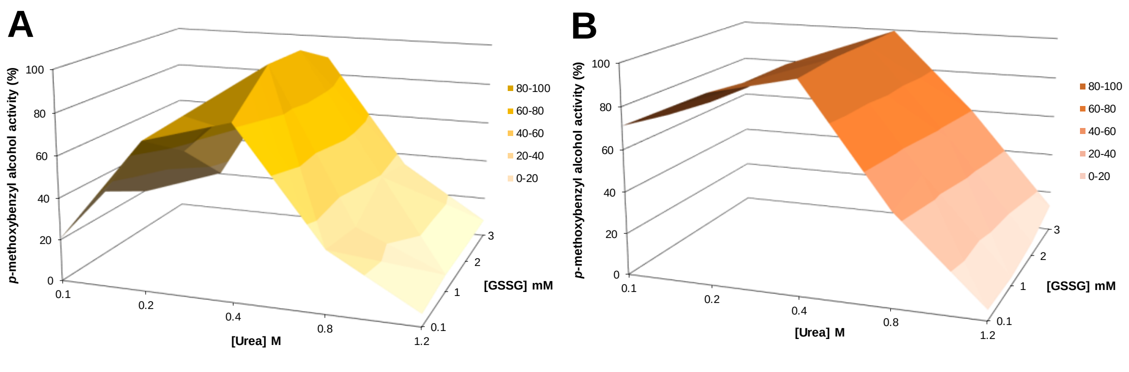

Figure S1. ***In vitro* activation of AAOs.** Optimization of urea and GSSG concentrations for *in vitro* refolding of *Po*AAO (**A**) and *Ba*AAO (**B**) determined in 96-well plate screening after 4-days incubation at 4 ºC. The figure shows the results obtained in 50 mM Tris/HCl supplemented with 200 µM FAD and 20% glycerol at pH 8.5 and pH 9.5 for *Po*AAO and *Ba*AAO, respectively.

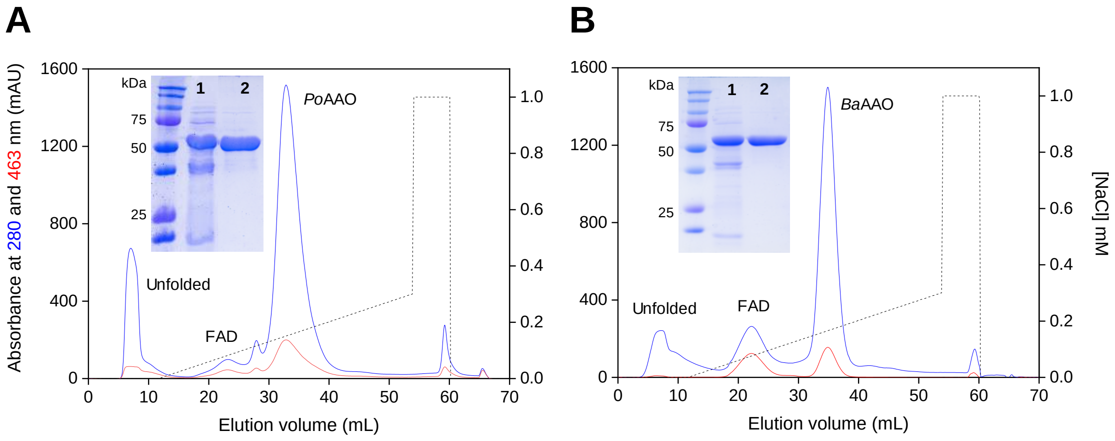

Figure S2. Purification of AAOs. ResourceQ chromatogram showing the elution profiles of *Po*AAO (**A**) and *Ba*AAO (**B**) at 280 (blue) and 463 nm (red) and the NaCl gradient (dashed line) with the corresponding SDS-PAGE of the refolding mixture (lanes 1) and the purified enzymes (lanes 2). Precision Plus Protein™ Dual Color Standards from BioRad was used as molecular weight marker.

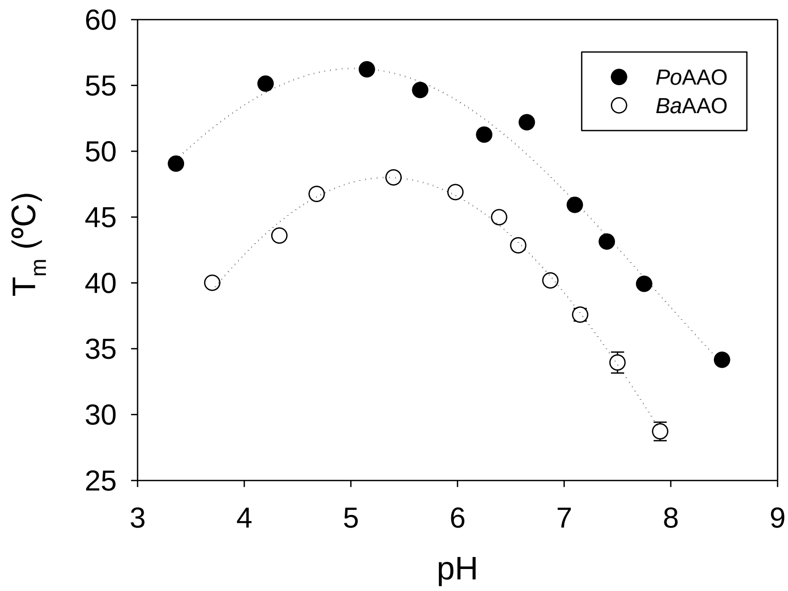

Figure S3. pH stability of *Po*AAO and *Ba*AAO. Melting temperature of *Po*AAO (closed circles) and *Ba*AAO (open circles) at different pHs in 100 mM B&R buffer.

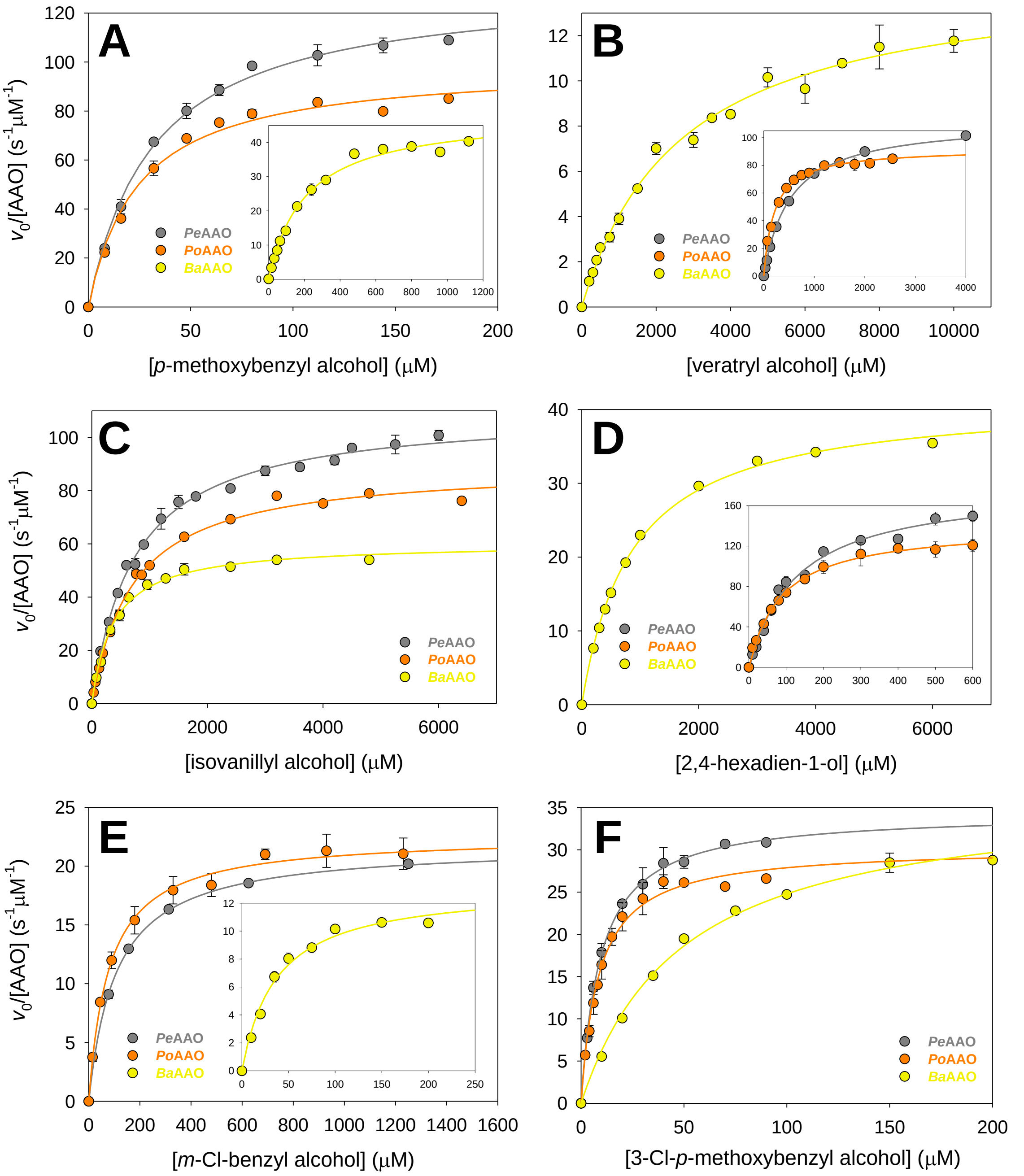

Figure S4. Steady-state kinetics. Comparison of steady-state kinetics for the oxidation of (**A**) *p*-methoxybenzyl alcohol, (**B**) veratryl alcohol, (**C**) isovanillyl alcohol, (**D**) 2,4-hexadien-1-ol, (**E**) *m*-Cl-benzyl alcohol and (**F**) 3-Cl-*p*-methoxybenzyl alcohol by *Pe*AAO (gray), *Po*AAO (orange) and *Ba*AAO (yellow) determined in air-saturated 50 mM NaPi, pH 6.0, at 25 ºC. The corresponding Michaelis-Menten fittings are shown as solid lines.

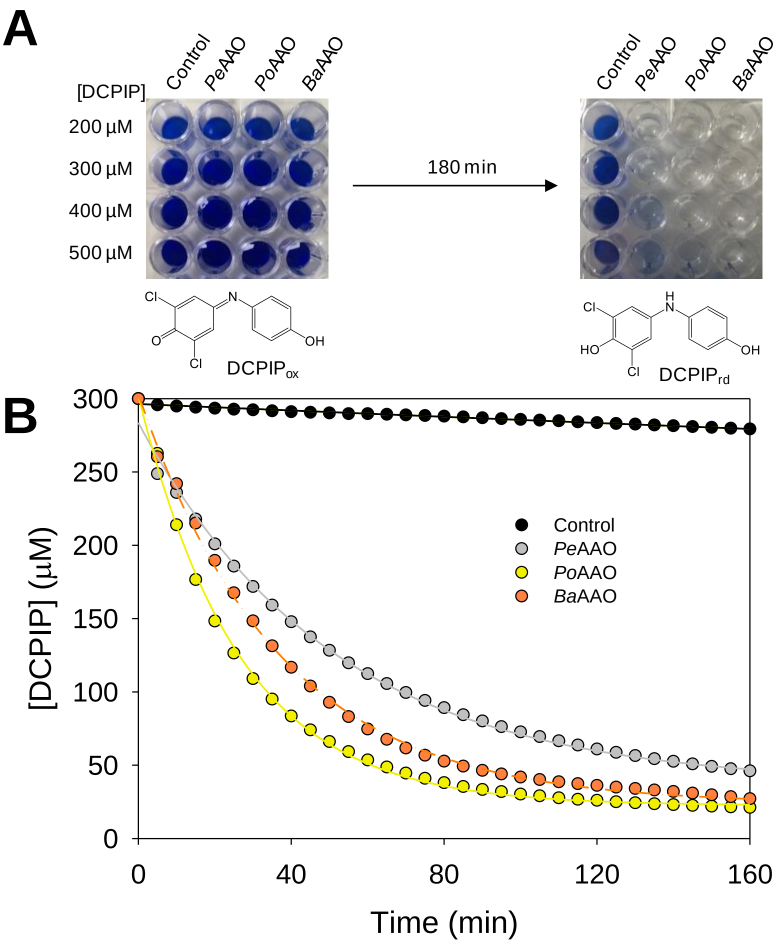

v_0/[AAO] (s^{-1})		
*Pe*AAO	*Po*AAO	*Ba*AAO
0.55 ± 0.04	1.30 ± 0.06	0.82 ± 0.03

Figure S5. DCPIP reduction. (**A**) Microtiter plate activity assay for *Pe*AAO, *Po*AAO and *Ba*AAO (0.1 µM) with different DCPIP concentrations. (**B**) Time course of the absorption change of 300 µM DCPIP with 0.1 µM enzymes and the sample control without enzyme. Reactions were performed in 50 mM NaPi, pH 6.0 at 25 ºC. Initial velocities (v_0/[AAO]) were obtained by fitting initial rates to a linear equation.

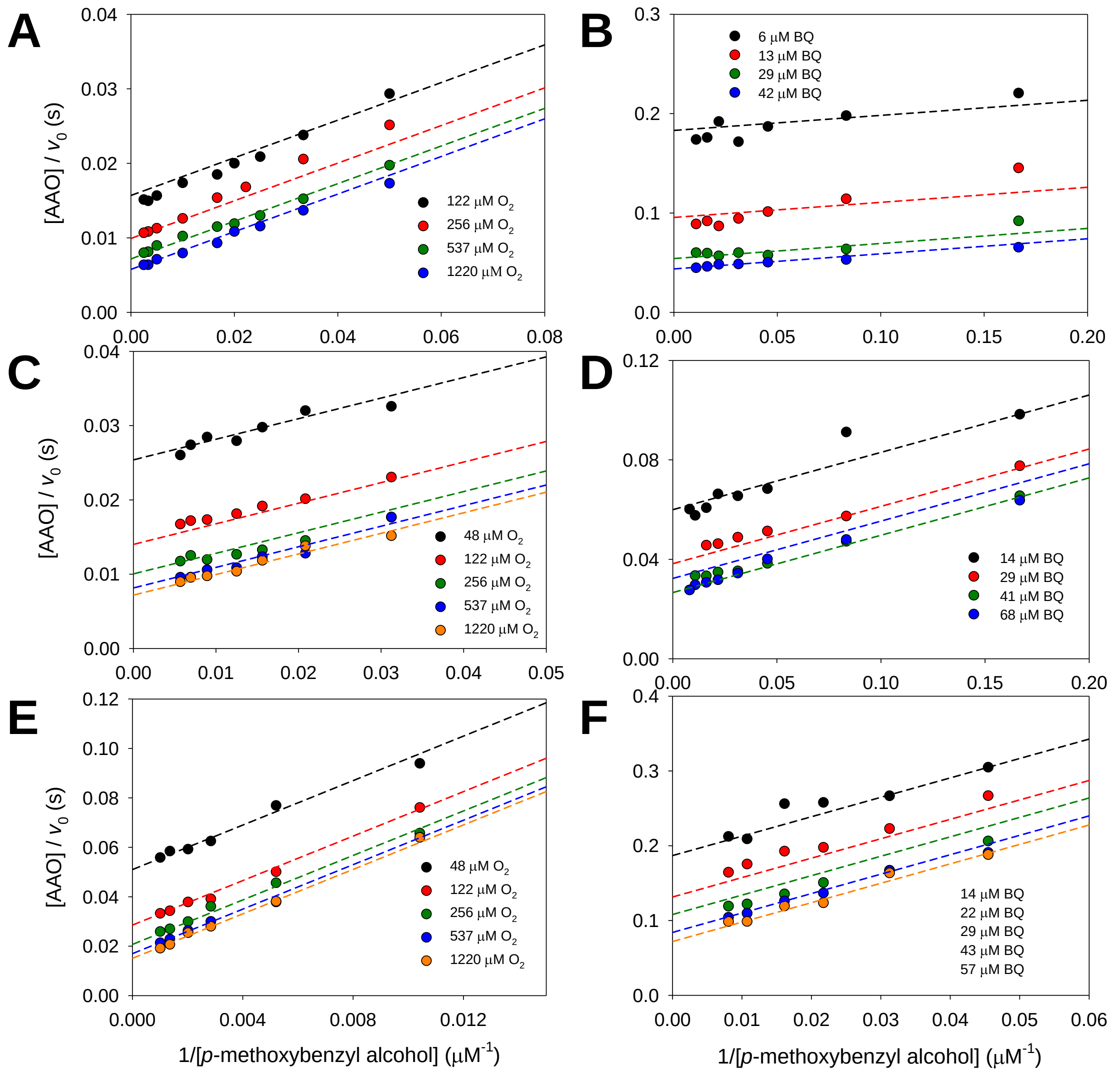

Figure S6. Bisubstrate kinetics for AAOs. Double reciprocal plots of the oxidation of *p*-methoxybenzyl alcohol by *Pe*AAO (**A** and **B**), *Po*AAO (**C** and **D**) and *Ba*AAO (**E** and **F**) with oxygen (**A**, **C** and **E**) and BQ (**B**, **D** and **F**) as electron acceptors.

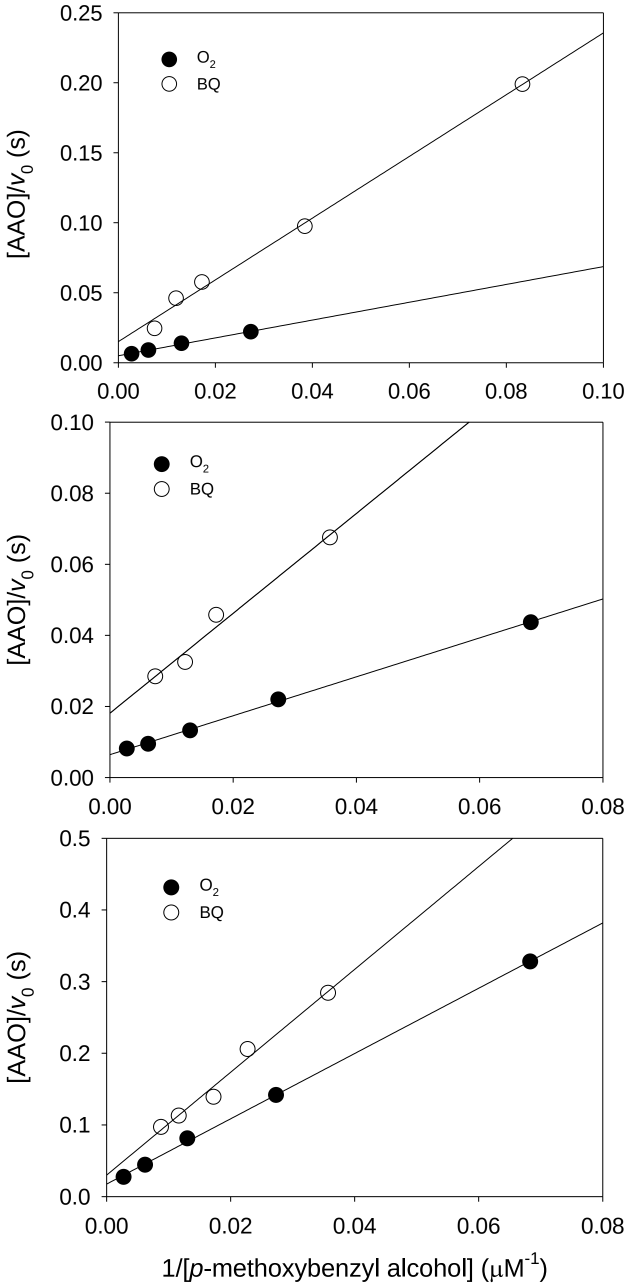

Figure S7. Double reciprocal plots of the initial rates of reaction as a function of the concentration of *p*-methoxybenzyl alcohol at a fixed ratio [alcohol]/[oxygen] of 0.3 (closed circles) or [alcohol]/[BQ] of 2 (open circles) for *Pe*AAO (A), *Po*AAO (B) and *Ba*AAO (C). Experimental data were fitted to Eq. 4 with R^2 values of 0.9963 and 0.9949 for *Pe*AAO with oxygen and BQ, respectively (panel A), of 0.9992 and 0.9786 for *Po*AAO with oxygen and BQ, respectively (panel B) and of 0.9995 and 0.9835 for *Ba*AAO with oxygen and BQ, respectively (panel C).

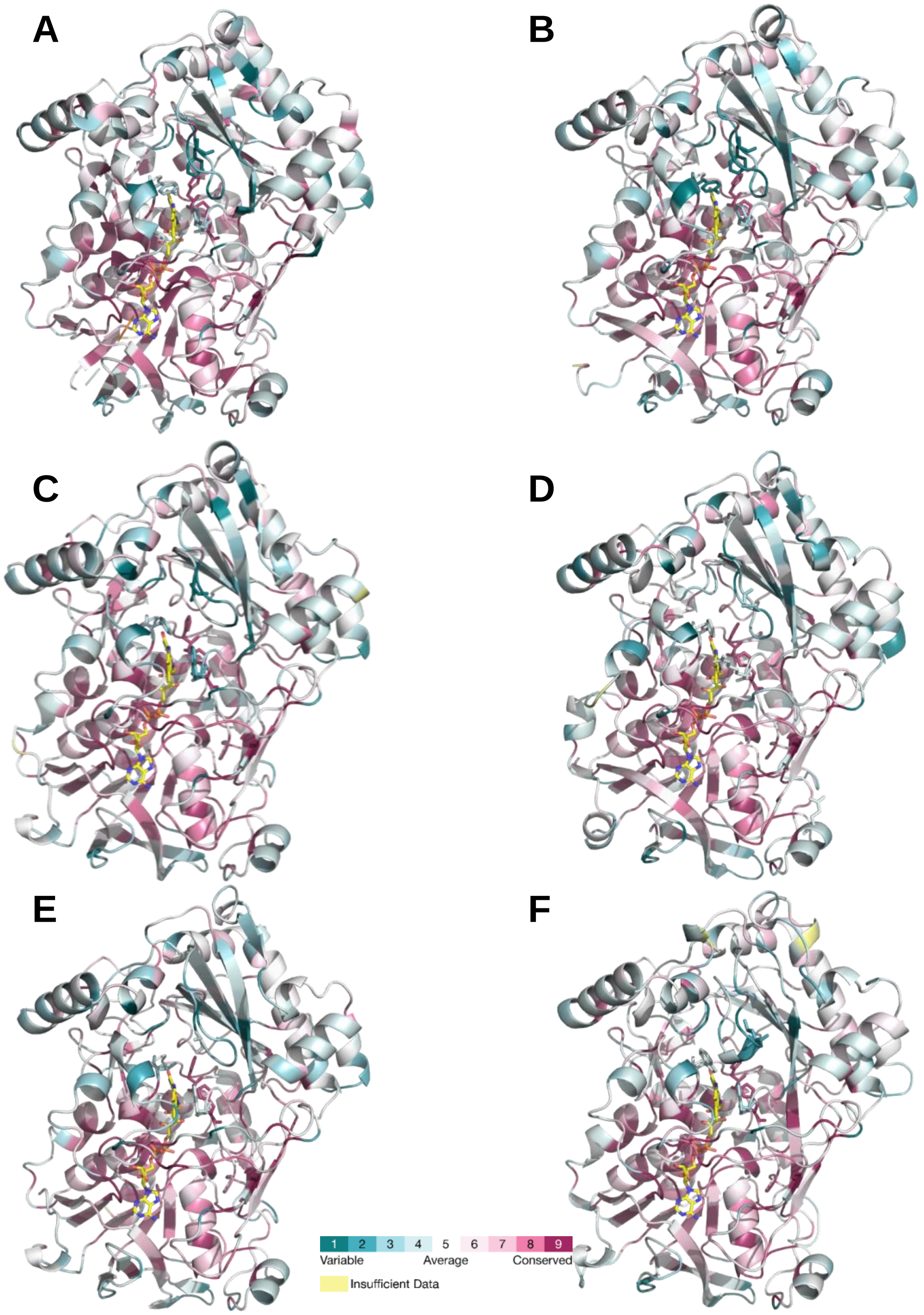

Figure S8. Overall secondary structure representations of AAOs and AADHs. (**A**) *Pe*AAO (from crystal structure 3FIM), (**B**) *Po*AAO, (**C**) *Ba*AAO, (**D**) *Pc*AADH1, (**E**) *Pc*AADH2 and, (**F**) *Pc*AADH3. Models were obtained using AlphaFold. FAD moiety is represented with sticks in CPK colors, with carbons in yellow and secondary structure is colored according to the conservation scores calculated by ConSurf server.

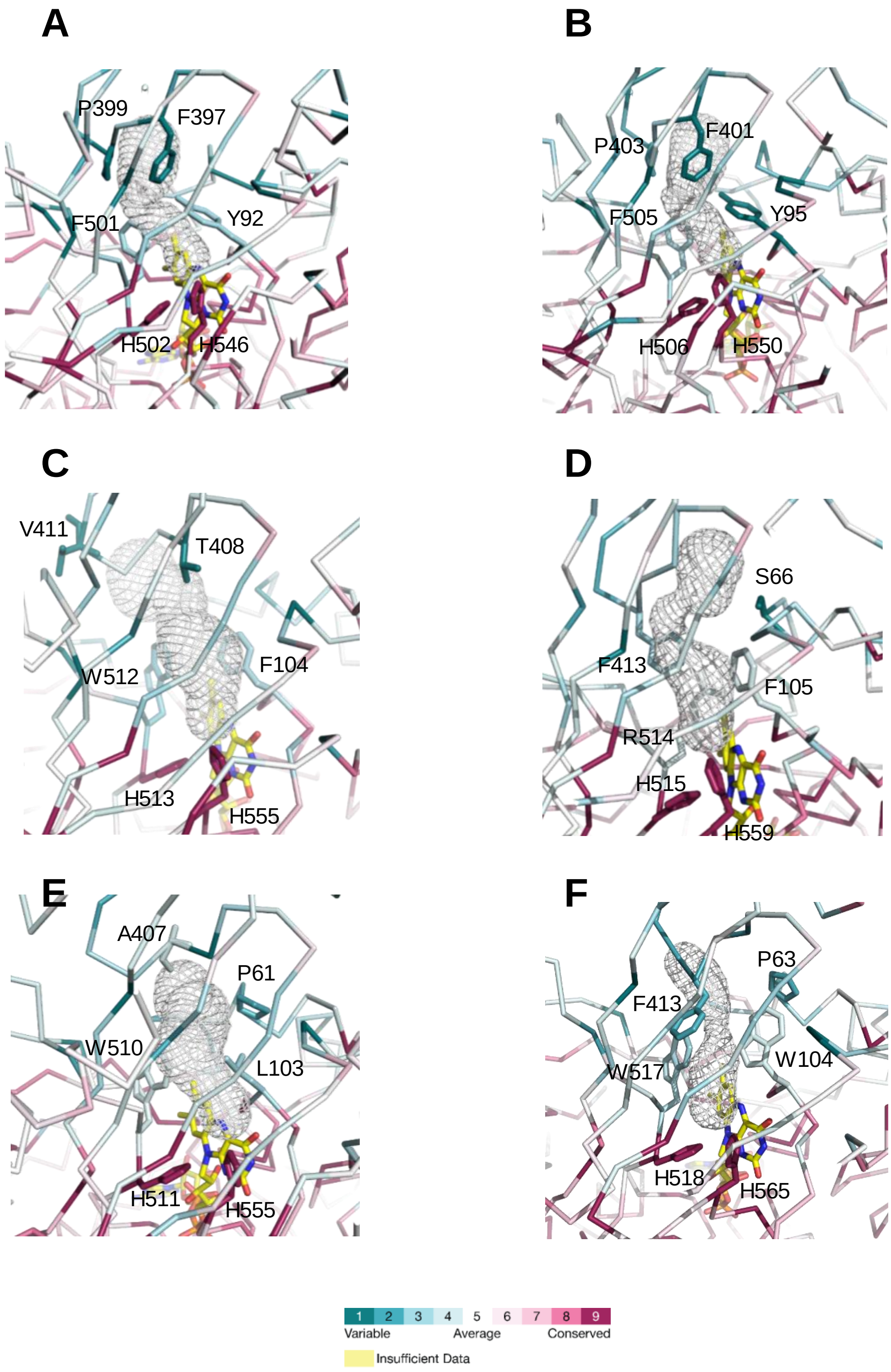

Figure S9. Detail of the active sites and access channels to the active site of AAOs and AADHs. (**A**) *Pe*AAO, (**B**) *Po*AAO, (**C**) *Ba*AAO, (**D**) *Pc*AADH1, (**E**) *Pc*AADH2 and, (**F**) *Pc*AADH3. The active-site access channels were depicted by CAVER. FAD moiety is represented with sticks in CPK colors, with carbons in yellow and catalytic residues and those involved in the substrate access channel formation are shown in sticks and colored according to the conservation scores calculated by ConSurf server.

Table S1. Comparison of steady-state kinetic parameters —k_{cat} (s^{-1}), K_m (μM), and k_{cat}/K_m ($mM^{-1}s^{-1}$)— for the oxidation of several alcohols by *Pe*AAO, *Po*AAO and *Ba*AAO determined in air-saturated 50 mM NaPi, pH 6.0, at 25ºC.

		*Pe*AAO	*Po*AAO	*Ba*AAO
p-methoxybenzyl	k_{cat}	132 ± 3	99 ± 3	48 ± 1
	K_m	32 ± 2	24 ± 2	207 ± 17
	k_{cat}/K_m	4120 ± 300	4100 ± 440	234 ± 14
veratryl	k_{cat}	112 ± 1	92 ± 1	15 ± 1
	K_m	528 ± 21	212 ± 11	2680 ± 210
	k_{cat}/K_m	213 ± 9	435 ± 24	6 ± 1
isovanillyl	k_{cat}	110 ± 1	90 ± 2	60 ± 1
	K_m	745 ± 34	713 ± 44	376 ± 25
	k_{cat}/K_m	148 ± 7	126 ± 8	160 ± 9
2,4-hexadien-1-ol	k_{cat}	119 ± 1	140 ± 2	42 ± 1
	K_m	94 ± 4	86 ± 4	854 ± 32
	k_{cat}/K_m	1270 ± 60	1630 ± 90	49 ± 1
vanillyl	k_{cat}	nd	nd	38 ± 1
	K_m	nd	nd	1960 ± 100
	k_{cat}/K_m	nd	nd	19 ± 1
m-chloro benzyl	k_{cat}	22 ± 1	23 ± 1	13 ± 1
	K_m	107 ± 2	80 ± 6	37 ± 5
	k_{cat}/K_m	203 ± 3	282± 21	358 ± 49
3-chloro-*p*-methoxybenzyl	k_{cat}	34 ± 1	30 ± 1	37 ± 1
	K_m	9 ± 1	9 ± 1	49 ± 4
	k_{cat}/K_m	3660 ± 150	3500± 280	749 ± 59

nd. not determined
